# Orthodontic Elastics: A Narrative Review of Biomechanics, Biological Responses, and Evidence-Based Clinical Guidelines for Everyday Practice

**DOI:** 10.7759/cureus.100972

**Published:** 2026-01-07

**Authors:** Alaa Gamal Hassan, Mohamed Elshial, Bahaa Hassan, Mohamed Ismail Ahmed, Eslam Nabil H Sabry

**Affiliations:** 1 Orthodontics, Align Pro Academy, Cairo, EGY; 2 Orthopaedics and Trauma, Hywel Dda University Health Board, Carmarthen, GBR; 3 Psychiatry, Norfolk and Suffolk NHS Foundation Trust, Norwich, GBR; 4 Orthodontics, Microscopia Dental Clinic, Alexandria, EGY; 5 Orthodontics, Dr. Eslam Nabil's Dental Clinic, Alexandria, EGY

**Keywords:** biomechanics, class ii elastics, class iii elastics, force decay, functional appliances, intermaxillary elastics, latex, non-latex, orthodontic elastics, temporary anchorage devices

## Abstract

Orthodontic elastics remain fundamental auxiliaries in both fixed appliance and clear aligner treatment, providing versatile intermaxillary and intra-arch force systems. Their clinical performance is determined by lumen diameter, material composition, elastic memory, and force decay behaviour, which together influence the periodontal ligament response and surrounding hard and soft tissues. Latex elastics generally provide higher initial force levels and superior elastic recovery but are associated with risks of mucosal irritation and hypersensitivity. Non-latex alternatives improve biocompatibility yet tend to show greater force degradation over time. Medium-force elastics (approximately 3.5-4.5 oz) are commonly used for sagittal correction, whereas lighter 2-3 oz configurations are preferred for vertical control, finishing, and intercuspation. The increasing integration of skeletal anchorage and aligner therapy allows more precise vector control and helps to minimise unwanted effects such as molar extrusion, occlusal plane rotation, and incisor tipping.

This narrative review summarises current evidence on the biomechanics, material properties, biological responses, and clinical applications of orthodontic elastics, and synthesises practical recommendations for everyday practice. A structured, non-systematic search of PubMed, Scopus, and Google Scholar identified relevant clinical trials, laboratory investigations, systematic reviews, and key textbook chapters on force delivery, force decay, biocompatibility, compliance, and clinical outcomes.

On the basis of the available evidence, light, continuous forces within the range of approximately 70-120 g, combined with at least twice-daily elastic replacement and rigorous attention to vector control, appear optimal for safe and efficient tooth movement. Material selection should balance mechanical stability with biocompatibility, particularly in patients with suspected latex sensitivity. Mastery of elastic biomechanics, awareness of biological limits, and proactive management of patient compliance together underpin predictable and patient-centred outcomes.

## Introduction and background

Orthodontic elastics are fundamental auxiliaries in contemporary orthodontic treatment and remain widely used across fixed appliance, aligner-based, and skeletal anchorage protocols. Their clinical utility lies in their ability to deliver controlled intermaxillary and intra-arch forces that address sagittal discrepancies, enhance vertical control, improve intercuspation, and aid midline correction. The mechanical behaviour of elastics, governed by lumen diameter, material composition, stretch ratio, and force decay, directly influences the magnitude, direction, and duration of force transmitted to the dentoalveolar structures [1,2]. Despite the rapid evolution of digital orthodontics and skeletal anchorage techniques, orthodontic elastics remain indispensable for fine-tuning occlusal relationships, managing sagittal and vertical discrepancies, and enhancing finishing stages in both fixed appliance and aligner-based systems. Their continued use in everyday clinical protocols underscores the need for an updated, evidence-based understanding of their biomechanical and biological behaviour.

Optimal orthodontic tooth movement requires light, continuous forces within a biologically tolerable range. Yet, elastics are subject to significant intraoral degradation, variable patient compliance, and material-dependent differences in force stability [3,4]. Latex elastics typically demonstrate superior elastic memory and more consistent force delivery but may provoke hypersensitivity or mucosal irritation, whereas non-latex alternatives offer improved biocompatibility with accelerated force decay [5]. The increasing integration of temporary anchorage devices and clear aligner therapy has further diversified elastic application, enabling more precise vector control with fewer unwanted effects such as molar extrusion, incisor tipping, or occlusal plane rotation [6-8].

Although elastics are widely utilised in everyday practice, their biomechanical effects, biological implications, and clinical indications are often presented in isolation. Few recent reviews have synthesised these domains into a unified clinical framework.

This narrative review aims to bridge this gap by summarising current evidence on the biomechanics, biological responses, material characteristics, and clinical applications of orthodontic elastics, and by providing practical guidelines to support safe, efficient, and evidence-based use in routine orthodontic practice.

Methods

A non-systematic literature search was performed across PubMed, Scopus, and Google Scholar between January and September 2025. The following search terms were used alone or in combination: orthodontic elastics, intermaxillary elastics, latex, non-latex, force decay, biomechanics, Class II elastics, Class III elastics, functional appliances, temporary anchorage devices (TADs), and aligner elastics. Eligible sources included clinical trials, laboratory studies, systematic reviews, relevant retrospective studies, and authoritative textbook chapters. No restrictions were placed on publication year. Reference lists of key papers were manually screened to identify additional relevant literature.

Articles were selected for inclusion according to their relevance to four predefined thematic domains: (i) biomechanics and force systems, (ii) material properties and force decay, (iii) biological responses and biocompatibility, and (iv) the clinical applications of elastics together with patient-reported outcomes. These domains were chosen to ensure that the review synthesised evidence across the mechanical, biological, and patient-centred aspects of elastic therapy.

Only English-language sources were included. Editorials, opinion pieces, and non-peer-reviewed material were excluded. As this is a narrative review, no quantitative synthesis or formal risk-of-bias assessment was carried out. The findings were synthesised qualitatively and integrated into practical clinical recommendations.

## Review

Classification and configuration of orthodontic elastics

Functional Classification

Orthodontic elastics can be broadly categorised into intra-arch elastics, which are applied within a single dental arch, and inter-maxillary elastics (IMEs), which transmit force between the maxillary and mandibular arches [9]. Intra-arch, or Class I, elastics are primarily used for space closure, segmental alignment, and anchorage reinforcement. IMEs, by contrast, are classified according to the direction and magnitude of the force vector they produce. Common clinical groups include (i) Class II elastics, extending from the mandibular molar or premolar to the maxillary canine, (ii) Class III elastics, applied in the reverse direction, (iii) cross elastics, used to correct transverse discrepancies, (iv) vertical “settling” elastics, which facilitate intercuspation, and (v) diagonal elastics, which aid in midline correction. Each configuration generates a distinct three-dimensional force system that influences sagittal, vertical, and transverse control. Careful biomechanical planning is therefore essential to minimise undesirable effects such as extrusion or arch rotation [10].

IME Configurations: Clinical Applications

**Figure 1:**
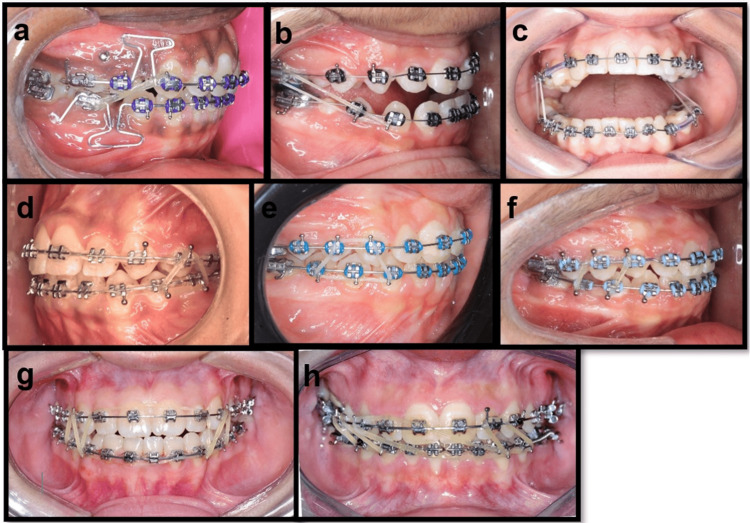
Representative clinical configurations of intermaxillary elastic patterns demonstrating sagittal, vertical, transverse, and asymmetry correction Variations in attachment points and vector direction create distinct three-dimensional force systems for targeted clinical effects. (A) Class II elastic: maxillary canine to mandibular molar; (B) Class III elastic: mandibular canine to maxillary molar; (C) Unilateral cross elastic with contralateral triangular settling elastic; (D) Posterior triangle settling elastic engaging premolar segments; (E) Trapezoid-pattern posterior settling elastic; (F) Posterior box elastic with a Class II vector; (G) Anterior box elastic with bilateral triangular settling elastics; (H) Anterior diagonal elastic for midline correction, combined with bilateral triangular settling elastics Image Credit: Authors; written informed consent was obtained from all patients for the use of their images.

Building on this classification, the clinical implementation of IMEs depends not only on their vector category but also on the choice of attachment points, the length of the elastic span, and the resulting direction of pull. These factors collectively determine the magnitude and orientation of the force transmitted to individual teeth or dental segments. In practice, elastics are used across a diverse range of applications, including Class II and Class III sagittal correction, unilateral crossbite management, vertical settling through triangle, box, or trapezoid patterns, midline or asymmetry correction with diagonal elastics, and combined anterior and posterior finishing mechanics. Even small modifications in hook position, anchorage preparation, or elastic direction can meaningfully alter the generated force vector, influencing both the intended movement and potential secondary effects on vertical or transverse control. These clinical configurations are illustrated in Figure 1.

Clinical Summary

IMEs provide highly adaptable biomechanics. By selecting the appropriate configuration and attachment points, clinicians can tailor force systems to address sagittal discrepancies, vertical finishing, transverse issues, and midline deviations. A proper understanding of force direction and magnitude is critical to maintaining control over occlusal plane changes, rotations, and unwanted extrusion.

Extra-oral elastics

Extra-oral elastics remain an essential component of orthopedic maxillary protraction, particularly in the early management of skeletal Class III malocclusion. Unlike intraoral elastics, these high-force systems act on the circummaxillary sutures rather than individual teeth, producing forward displacement of the maxilla and downward-backwards rotation of the mandible [11,12].

Biomechanics of Maxillary Protraction

Facemask elastics are typically applied bilaterally from vestibular hooks on a bonded maxillary splint to the adjustable crossbar of the facemask at an approximate 30° downward and forward vector. This orientation enables an anteriorly directed orthopedic force on the maxilla while controlling vertical effects. Therapeutic forces of ~400 g per side are commonly used during the active protraction phase, generating a total orthopedic load of approximately 800 g, which is required for clinically meaningful maxillary advancement [11,12].

Force Levels and Clinical Protocol

Randomised clinical trials by Mandall and colleagues describe a progressive elastic sequence to achieve effective orthopedic stimulation during facemask therapy [11,12]. Elastic dimensions and force ratings used in this protocol are summarised in Table 1.

**Table 1:** Extra-oral elastics used in protraction facemask therapy Data extracted from: Mandall et al., 2010 [11]; Mandall et al., 2012 [12] Clinical note: Elastics are applied bilaterally from the maxillary splint to the facemask crossbar, delivering a total orthopedic load of approximately 800 g at a 30° downward-forward angle

Elastic Size (inch)	Force Rating (oz)	Approx. Force (g per side)	Clinical Purpose / Phase
3/8″	8 oz	~225 g	Initial traction (1–2 weeks): light orthopedic stimulus
1/2″	14 oz	~400 g	Intermediate phase for maxillary protraction
5/16″	14 oz	~400 g	Progression to full orthopedic loading with downward–forward vector

Elastic dimensions, force calibration, and line of action

Lumen Diameter and Force Calibration

Orthodontic elastics are manufactured in standard lumen diameters ranging from 1/8 inch (≈3 mm) to 3/8 inch (≈10 mm). The delivered force is calibrated by stretching the elastic to three times its original diameter, at which point the nominal force rating is achieved. Force categories are typically standardised as very light, light, medium, heavy, and very heavy, corresponding approximately to 14-340+ grams of force [9]. Smaller lumen diameters generate higher initial forces for a given elongation but may fatigue more rapidly under repeated cyclic loading. Larger diameters provide lower initial force with a gentler decay profile.

Table 2 summarises typical lumen diameters and their common clinical indications, while Table 3 outlines the corresponding force categories when elastics are stretched to three times their original diameter.

**Table 2:** Typical internal diameters of intraoral orthodontic elastics Adapted from: Naini and Gill, 2022 [9] Clinical note: Elastic size determines both the stretch potential and the rate of force decay. Smaller diameters deliver greater initial force for a given elongation, but fatigue faster under repeated stretching

Lumen Diameter (inch)	Approximate Diameter (mm)	Common Clinical Indications
1/8″	3 mm	Fine vertical or finishing elastics; minimal interarch distance.
3/16″	4.8 mm	Standard Class II/III correction; most common universal size.
1/4″	6.4 mm	Heavier Class II correction or use with aligners; mild skeletal anchorage cases.
5/16″	8.0 mm	Long-span elastics, e.g., facemask or skeletal protraction.
3/8″	9.5–10 mm	Rarely used intraorally; typically for extra-oral or orthopedic traction.

**Table 3:** Typical force ranges of intraoral orthodontic elastics (stretched to three times original diameter) Clinical note: Orthodontic elastics are typically calibrated at three times their lumen diameter. Exceeding this elongation increases initial force output but accelerates force decay and material fatigue. Adapted from: Naini and Gill, 2022 [9]

Force Category	Force (Ounces)	Force (Grams)	Common Clinical Application
Very Light	0.5–1 oz	14–28 g	Early alignment, minimal interarch forces.
Light	2–5 oz	57–142 g	Finishing, intercuspation, mild vertical correction.
Medium	6–10 oz	170–283 g	Routine Class II/III correction; standard intermaxillary traction.
Heavy	11–12 oz	312–340 g	Short-duration heavy traction (skeletal anchorage-assisted).
Very Heavy (Orthopedic)	>12 oz	>340 g	Orthopedic force levels—facemask or BAMP therapy.

Points of Force Application

Elastic forces may be applied through several types of attachments, including hooks integrated into brackets or molar tubes, auxiliary hooks or loops incorporated into archwires, and Kobayashi ligatures used for engaging anterior teeth. Skeletal anchorage systems such as TADs or miniplates may also serve as stable points of elastic engagement. The choice of attachment point determines the vertical and horizontal components of the resulting force vector and therefore influences whether the resulting tooth movement is predominantly translational, rotational, or a combination of both [7,10,13].

Line of Action and Centre of Resistance

**Figure 2:**
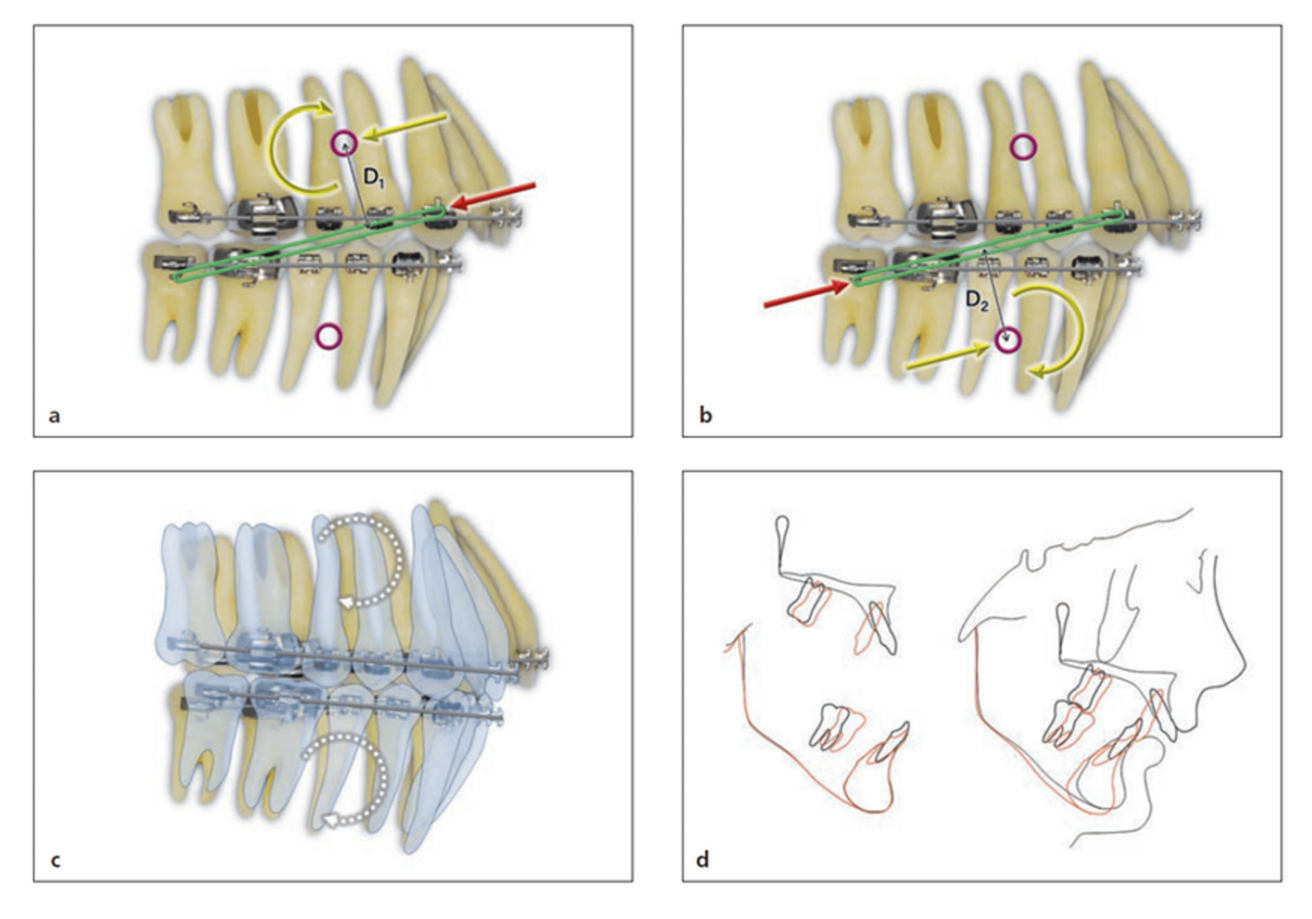
Long Class II elastic (a and b) A single force from the elastic (red arrow) is replaced with an equivalent force system (yellow arrows) at the CRs of the maxillary and mandibular arches. (c) Both arches rotate synchronously (dotted curved arrows) in the same clockwise direction because of the same magnitude and direction of the moments (D1 = D2). (d) Lateral superimposition of cephalometric radiographs before (black) and after (red) long-term use of Class II maxillomandibular elastics in an extraction case using round wire. Both the maxillary and mandibular arches rotated in a clockwise direction, with extrusion of the maxillary anterior teeth and mandibular posterior teeth. Image Source: Choy and Burstone, *Burstone’s Biomechanical Foundation of Clinical Orthodontics*, 2022 [13]; Published with permission from the copyright holder, Quintessence Publishing Co Ltd., Chicago, United States

**Figure 3:**
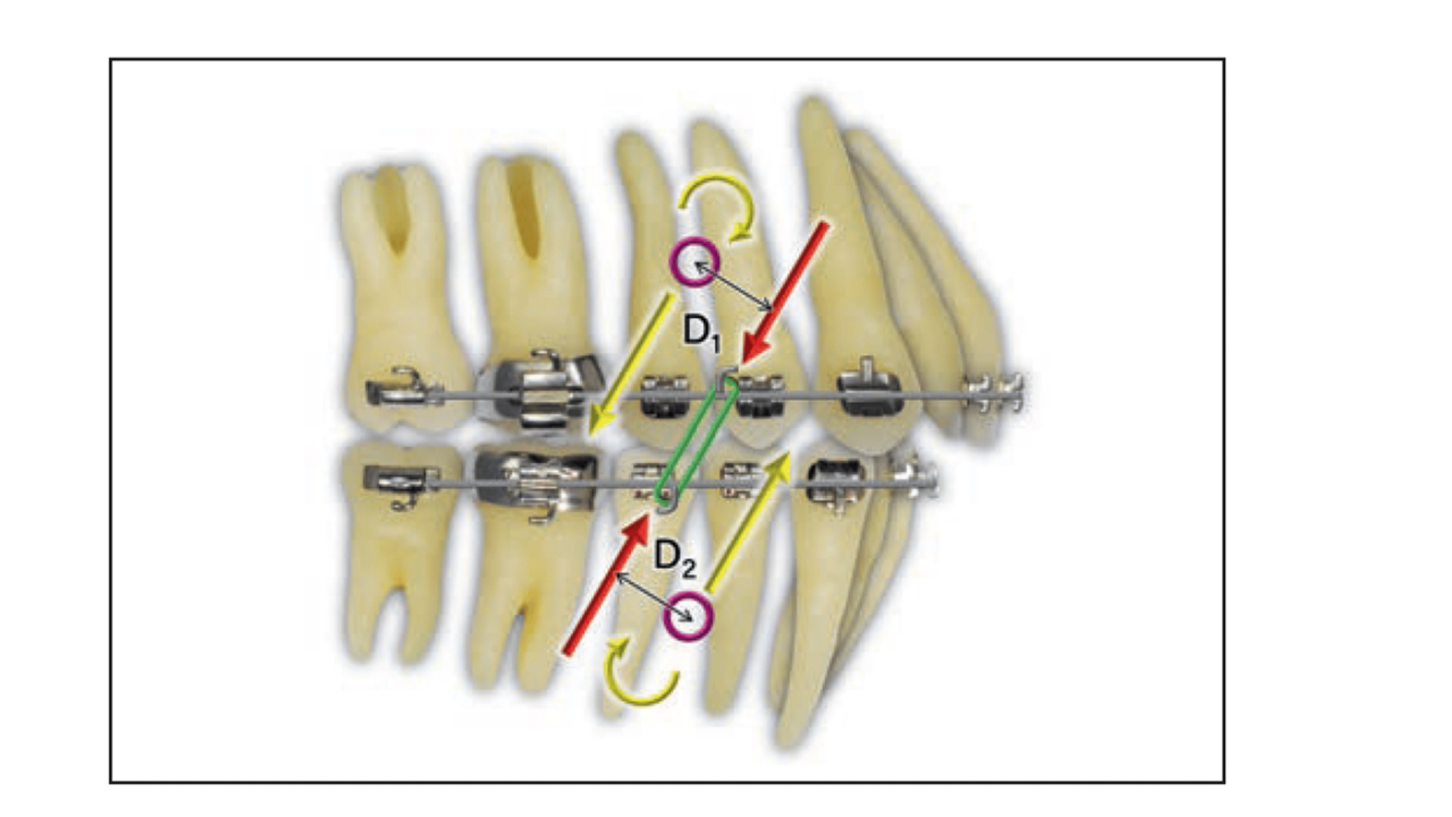
Short Class II elastic A single force from the elastic (red arrows) is replaced with an equivalent force system (yellow arrows) at the CR of each arch. It is also synchronous because the CR is an equal distance from the force in each arch (D1 = D2). The moment is lower and the vertical component of force is greater than with the long Class II elastic. CR: centre of resistance Image Source: Choy and Burstone, *Burstone’s Biomechanical Foundation of Clinical Orthodontics*, 2022 [13]; Published with permission from the copyright holder, Quintessence Publishing Co Ltd., Chicago, United States

**Figure 4:**
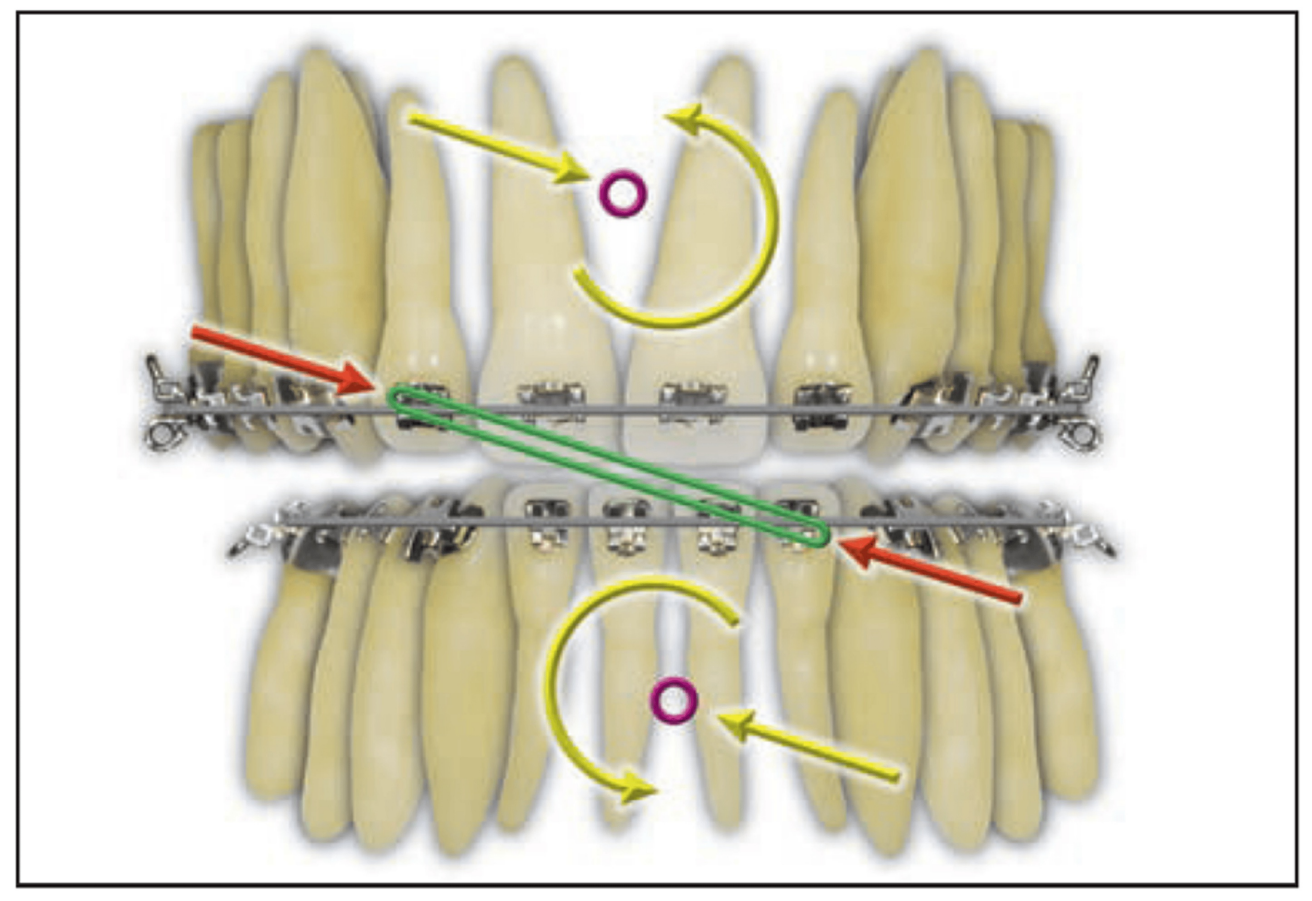
Anterior crisscross elastic (red arrows). Equivalent force systems at the maxillary and mandibular CRs are represented by yellow arrows. The force system would aid in midline correction; however, the canting from the moments of the maxillary and mandibular occlusal planes is undesirable. CR: centre of resistance Image Source: Choy and Burstone, *Burstone’s Biomechanical Foundation of Clinical Orthodontics*, 2022 [13]; Published with permission from the copyright holder, Quintessence Publishing Co Ltd., Chicago, United States

The biomechanical effect of an elastic is determined by the relationship between its line of action and the centre of resistance (CR) of the tooth, group of teeth, or dental arch to which it is applied. According to Burstone’s classical analysis, when the line of force passes directly through the CR, the resulting movement is predominantly bodily with minimal rotation [13]. When the line of action is displaced from the CR, a moment is generated, where the magnitude of this moment (M) is expressed as the product of the applied force (F) and the perpendicular distance (d) from the line of action to the CR. This offset produces rotational effects, arch tipping, or changes in the occlusal plane. Asymmetrical or diagonal vectors introduce additional differential moments, which may result in transverse rotation or arch canting unless carefully balanced or alternated. These biomechanical principles are illustrated in Figures 2-4.

Clinical Interpretation

The choice of elastic configuration must therefore account not only for sagittal vector correction but also for the secondary vertical and transverse effects introduced by the line of action. Shorter vertical spans (short Class II/III mechanics) minimise unwanted rotation, while longer spans increase vertical side effects such as molar extrusion or occlusal plane alteration.

Mechanical properties and force decay

The clinical performance of orthodontic elastics is determined by their initial force delivery, elastic memory, and resistance to force decay under intraoral conditions. As orthodontic tooth movement depends on light, continuous forces, understanding the degradation behaviour of elastic materials is essential for maintaining biologically effective loading [3,4,14].

Force Delivery and Decay Dynamics

When stretched to three times their lumen diameter, elastics deliver their nominal force. However, intraoral factors, such as 37°C temperature, humidity, saliva, and pH fluctuations, lead to stress relaxation and time-dependent loss of force [15].

Patterns of force decay follow a characteristic sequence in orthodontic elastics. There is a rapid initial loss of tension during the first hour of wear, after which the decline becomes more gradual. Repeated stretching during mastication further increases force instability, and over time, the applied forces often drift outside the biologically ideal 70-120 g range required for light, continuous tooth movement [3,4,16]. Material differences also contribute to degradation behaviour, with latex elastics maintaining higher residual force levels over comparable intervals, whereas non-latex elastics demonstrate greater early and cumulative decay [14,17,18].

Elastic Memory and Permanent Deformation

Elastic memory, the ability of a material to return to its original length following stretching, is generally superior in natural latex (cis-1,4-polyisoprene). Latex elastics exhibit rapid elastic recovery, minimal permanent deformation, and more consistent force delivery across repeated activation cycles [19]. In contrast, synthetic non-latex materials such as polyurethane or synthetic polyisoprene tend to undergo greater plastic deformation, display reduced elastic recovery, and show increased permanent elongation during wear [18]. These material differences account for the common clinical observation that non-latex elastics feel “softer” during use and require more frequent replacement.

Factors Influencing Force Decay

Several intrinsic and extrinsic factors influence the rate at which orthodontic elastics lose force. Elevated intraoral temperature increases molecular relaxation within the material, thereby reducing tensile strength, an effect that is particularly pronounced in latex elastics [15]. Humidity and saliva also contribute to degradation, as water absorption leads to swelling of the polymer matrix and increased chain slippage, both of which accelerate force decay [14]. Mechanical fatigue generated by mastication, together with fluctuations in oral pH, especially exposure to acidic beverages or poor oral hygiene, further weakens elastic polymers [20]. Storage conditions play an additional role; exposure to heat, light, or chemical disinfectants can degrade the polymer structure, whereas cold, dry, and dark environments help preserve material stability [21,22]. Environmental factors such as cigarette smoke and electromagnetic radiation have likewise been shown to alter the mechanical properties of elastics and hasten their degradation [23,24].

Comparative Properties of Latex and Non-Latex Elastics

Latex elastics generally demonstrate higher initial force levels, better force retention over 24 hours, less permanent deformation, and a comparatively rougher surface texture, although they are associated with a recognised risk of allergic reaction. Non-latex elastics, in contrast, typically show lower initial force, more rapid and continuous force decay, smoother surface characteristics, greater overall biocompatibility, and a tendency toward increased permanent elongation during wear. While latex remains biomechanically advantageous in most situations, non-latex alternatives are essential for patients with suspected or confirmed latex hypersensitivity. These mechanical, biological, and surface differences are summarised in Table 4, based on evidence from both in vitro and in vivo investigations [5,25-28].

**Table 4:** Comparative properties of latex and non-latex orthodontic elastics SEM: scanning electron microscope

Parameter	Latex Elastics	Non-Latex Elastics (Synthetic / Polyurethane)	Key References
Initial Force	Comparable or slightly higher initial force; more consistent across brands.	Similar or slightly lower initial force; greater variability by brand.	López et al., 2018 [25]; Malanon et al., 2019 [26]
Force Decay/Retention (24 hours)	Retains ~65–75% of initial force after 24 hours (better stability).	Greater force loss, retaining ~40–60% after 24 hours.	Klabunde and Grünheid, 2021 [27]; Notaroberto et al., 2018 [29]; López et al., 2012) [25]
Elastic Memory/Deformation	Smaller and more uniform deformation; maintains original shape better.	Greater deformation and permanent elongation over time.	Oliveira et al., 2017 [28]
Biocompatibility/Cytotoxicity	Can trigger allergic reactions (natural rubber proteins); slightly more cytotoxic in vitro.	Hypoallergenic alternative; higher cell viability and less irritation.	Martínez-Colomer et al., 2016 [5]; Lacerda-Santos et al., 2015 [30]
Surface Characteristics	Rougher, more porous surface (microspheres visible on SEM); more tissue irritation initially.	Smoother surface with crystal-like texture and fewer pores.	Martínez-Colomer et al., 2016 [5]
Force Behavior Under Dynamic Conditions	Rapid initial decay (mainly first hour), then stabilizes; performs better under cyclic load.	Continuous and higher decay across time; significantly weaker under cyclic conditions.	Klabunde and Grünheid, 2021 [27]; Kersey et al., 2009 [31]
Best Clinical Use	Preferred when mechanical consistency and strong force delivery are required; short-term wear.	Suitable for patients with latex hypersensitivity, long-term wear, or aesthetic requirements.	Synthesized from above evidence.

Biocompatibility and Hypersensitivity

Latex elastics are produced from natural rubber (cis-1,4-polyisoprene) containing water-soluble proteins, sulphur compounds, and antioxidants, any of which may act as antigens capable of provoking hypersensitivity reactions [32,33]. Two principal forms of allergy have been described. Type I IgE-mediated hypersensitivity presents rapidly and may cause mucosal erythema, swelling, pruritus, or, in rare cases, systemic manifestations. Type IV delayed cell-mediated hypersensitivity appears more gradually and is typically characterised by localised inflammation, itching, or contact dermatitis. Reported prevalence among orthodontic patients ranges from 1% to 6%, with higher rates observed in individuals with repeated medical or occupational latex exposure. Non-latex elastics, such as those made from polyurethane or synthetic polyisoprene, exhibit markedly greater biocompatibility and eliminate the risk of protein-mediated allergy [17,18]. For this reason, they are preferred in patients with a known history of latex sensitivity, in atopic individuals, and in cases requiring prolonged elastic wear. Routine clinical screening should therefore include a brief allergy history, including prior reactions to rubber gloves, balloons, or other latex-containing medical products.

Cytotoxicity and Cellular Response

Cytotoxicity studies conducted on fibroblast, epithelial, and neuronal cell lines consistently demonstrate material-dependent differences in cellular tolerance [5,30,34,35]. Latex elastics tend to exhibit mild to moderate cytotoxicity, particularly within the first 24 hours of exposure, a response attributed to the leaching of sulphur-based accelerators, zinc oxide, pigments, and other processing additives incorporated during manufacturing [34,35]. In contrast, non-latex elastics generally show substantially lower cytotoxic potential, with cellular viability frequently exceeding 90% and only minimal inflammatory infiltration observed in both in vitro and in vivo evaluations [5,30].

Clinical Implications

Transient mucosal irritation associated with latex elastics is most commonly linked to mechanical friction against the oral tissues, the release of chemical additives during the early phase of wear, or the prolonged retention of fatigued elastics that have degraded intraorally [5,34,35]. When such irritation occurs, it is typically managed by switching to non-latex alternatives or by increasing the frequency of elastic replacement to minimise exposure to degraded material [5,30].

Clinical guidelines for elastic selection and use

The successful application of orthodontic elastics depends on the controlled delivery of force in accordance with biomechanical principles, biological limits, and individual patient characteristics. Because elastics are patient-dependent auxiliaries, clinical decisions must integrate force magnitude, vector planning, vertical and transverse effects, and anticipated compliance.

Principles of Force Application

The biological response to orthodontic force is dose-dependent yet non-linear, with the most favourable tissue modelling achieved under light, continuous forces [3,4,16]. Forces within the range of 70-120 g are considered biologically safe for periodontal tissues, promoting effective periodontal ligament remodelling while minimising hyalinisation, whereas heavier or intermittent forces exceeding 200 g increase the likelihood of tissue damage, slow the rate of tooth movement, and elevate the risk of root resorption. Because the nominal force of an elastic is calibrated when the material is stretched to three times its original lumen diameter [9], maintaining biologically effective loading requires careful consideration of elastic size, material type, and replacement frequency. Equally important is an awareness of the vertical and transverse effects generated by different configurations, ensuring that force systems remain both controlled and physiologically tolerable.

Force Selection by Configuration

Force magnitude must be matched carefully to the intended tooth movement and the three-dimensional vector produced by the selected elastic configuration. In sagittal correction, such as Class II or Class III mechanics, medium-force elastics of approximately 4-6 oz generally provide sufficient intermaxillary traction while remaining within biologically acceptable limits. Vertical settling mechanics, including box or triangle elastics, require lighter forces of roughly 2.5-4.5 oz to minimise the risk of unwanted extrusion or alteration of the occlusal plane. Crossbite and diagonal elastics are typically applied using light-to-medium force levels to exert controlled transverse effects without inducing occlusal canting, whereas asymmetric mechanics should be applied with the minimum force necessary to prevent imbalance in the occlusal plane or midline deviation. Ultimately, elastic selection must be customised according to the patient’s skeletal pattern, anchorage requirements, and the anticipated vertical or transverse side effects of the chosen configuration. The recommended elastic sizes and force ranges for these clinical scenarios are summarised in Table 5, derived from classical biomechanical principles described by Burstone and standard clinical practice [13].

**Table 5:** Biomechanical configurations, effects, and suggested force parameters of intermaxillary elastics Adapted from: Choy and Burstone, 2022 [13]

Elastic Type/Configuration	Direction of Force Application	Line of Action Relative to Centers of Resistance	Primary Biomechanical Effects	Suggested Elastic Size	Typical Force Range, oz (g)	Clinical Considerations
Class II Elastic (Long Configuration)	From mandibular molar to maxillary canine	Passes above mandibular and below maxillary centers of resistance	Produces clockwise rotation of both arches; lower molar extrusion; occlusal plane steepening	1/4″ medium	4.5–6 oz (128–170 g)	May increase lower facial height; requires anchorage control and vertical management
Class II Elastic (Short Configuration)	Same sagittal direction but shorter vertical span	Passes closer to both centers of resistance	Reduces rotational moments; maintains occlusal plane stability	3/16″ medium	4–4.5 oz (113–128 g)	Preferred when vertical control is critical; minimizes bite opening
Class III Elastic (Long Configuration)	From mandibular canine to maxillary molar	Passes below maxillary and above mandibular centers of resistance	Produces counterclockwise rotation	1/4″ medium	4–6 oz (113–170 g)	Used for correction of anterior crossbite; monitor vertical changes
Class III Elastic (Short Configuration)	Reverse sagittal direction with shorter lever arms	Force vector closer to centers of resistance	Reduces moment generation; maintains vertical dimension	3/16″ medium	4–5 oz (113–142 g)	Improves control of mandibular plane rotation in Class III cases
Vertical (Box or Triangle) Elastics	Between corresponding teeth vertically in posterior or anterior regions	Force directed perpendicular to occlusal plane	Enhances intercuspation; facilitates vertical settling and bite closure	1/8″ or 3/16″ light	3–4.5 oz (85–128 g)	Useful during finishing phase; avoid excessive extrusion in high-angle cases
Cross Elastic	From buccal surface of one arch to lingual of opposing arch	Oblique vector crossing midline; asymmetric line of action	Produces rotation and transverse correction toward elastic side; may induce occlusal canting	3/16″ light	2.5–4.5 oz (70–128 g)	Used for unilateral or single-tooth crossbite correction; alternate sides to maintain balance
Subdivision / Asymmetric Elastic	Applied unilaterally between arches	Unequal vectors right vs. left	Corrects midline deviation or asymmetric molar relationships	3/16″ light–medium	3–4.5 oz (85–128 g)	Requires torque control to avoid canting; monitor midline alignment
Segmental Elastic (Buccal Segment Use)	Applied to localized segment rather than entire arch	Force limited to selected teeth	Achieves localized crossbite or AP correction with reduced unwanted vertical effects	3/16″ light	2.5–4 oz (70–113 g)	Minimizes side effects on anchorage; allows targeted biomechanical control

Duration and Frequency of Wear

Clinical evidence consistently demonstrates that full-time wear of approximately 20-22 hours per day results in the most predictable orthodontic correction, whereas intermittent or inconsistent use is strongly associated with treatment delay. To counteract the rapid force decay that occurs during the first hours of wear, elastics should be replaced twice daily, typically in the morning and evening [14,27]. Patients should also be encouraged to carry spare elastics to avoid unplanned lapses in wear, and correct placement should be reinforced regularly by having the patient demonstrate insertion during each visit. In most cases, failures in sagittal correction arise from insufficient compliance rather than from mechanical limitations of the elastics themselves.

Considerations for Different Skeletal Patterns

The vertical and rotational effects produced by IMEs differ according to the patient’s facial morphology, making it essential to modify mechanics based on skeletal pattern. In high-angle (hyperdivergent) cases, steep Class II vectors or long vertical spans should be avoided because they increase the risk of molar extrusion and clockwise mandibular rotation; shorter Class II or Class III configurations, or mechanics supported by temporary anchorage devices, are generally preferred to enhance vertical control. In contrast, low-angle (hypodivergent) patients may benefit from the mild extrusion associated with elastic wear, and vertical elastics can assist in normalising the curve of Spee and improving intercuspation. When using Class III camouflage mechanics, reverse elastics must be applied cautiously to prevent counterclockwise mandibular rotation and undesirable retroclination of the lower incisors, whereas skeletal anchorage systems such as bone-anchored maxillary protraction (BAMP) offer more stable skeletal correction with reduced dental side effects [7]. Overall, the selection of elastic configuration should take into account the occlusal plane, anchorage demands, and the anticipated vertical effects to ensure safe and predictable outcomes.

Compliance and patient-reported outcomes

The biomechanical effectiveness of orthodontic elastics is well established; however, clinical outcomes are fundamentally governed by patient compliance. Because elastics are removable and patient-controlled, variability in wear duration, replacement frequency, and technique represents the primary source of treatment inefficiency. Understanding the behavioural, psychological, and comfort-related factors that influence adherence is therefore essential for predictable outcomes.

Role of Compliance in Treatment Success

Sagittal correction with IMEs depends heavily on consistent full-time wear, typically 20-22 hours per day. Objective monitoring studies indicate that patients often overestimate their level of adherence, and intermittent use tends to produce temporary tipping movements rather than the stable translational effects required for meaningful correction [10]. Behavioural strategies have demonstrated measurable improvements in cooperation; for example, text-message reminders have been shown to increase daily wear time and accelerate Class II correction [36], while structured “if-then” behavioural planning can further enhance adherence, although clinical efficiency may still vary among individuals [37]. Collectively, these findings reinforce that patient compliance, far more than the mechanical properties of the elastics, is the primary determinant of successful treatment outcomes.

Psychological and Behavioural Factors

Motivation, perceived treatment benefit, and a clear understanding of the function of elastics all play important roles in determining patient cooperation. Egolf et al. (1990) demonstrated that motivation and perceived aesthetic improvement were among the strongest predictors of adherence [38], while Bartsch et al. (1993) noted that adolescents often show fluctuating wear patterns influenced by school schedules, peer perceptions, and the level of parental supervision [39]. Strategies that enhance cooperation include providing a clear explanation of the purpose of elastics and the anticipated progress, offering regular positive reinforcement, involving parents when treating younger patients, delivering structured feedback at each appointment, and ensuring that placement techniques are demonstrated repeatedly until the patient can perform them confidently. Notably, the quality of communication between clinician and patient appears to exert a greater influence on adherence than digital reminders alone

Patient-Reported Outcomes: Comfort, Pain, and Adaptation

Patient-reported comfort has a substantial influence on long-term compliance with elastic wear, as several short-term symptoms may appear during the initial adjustment period. Mild pain or tightness is common within the first 24-48 hours because of periodontal ligament compression, and some patients experience temporary difficulty with chewing or minor occlusal interference. Soft-tissue irritation of the cheeks or lips may arise from hooks or repeated contact, while occasional elastic breakage can occur when elastics are overstretched. In addition, transient mucosal irritation may result from latex additives. These symptoms typically improve as the patient adapts to treatment. Uzunçıbuk et al. (2023) reported that Class III elastics may cause greater early discomfort than Class II elastics, although both groups demonstrated significant improvement after several months [40]. Similarly, Nalamliang and Thongudomporn (2023) found that consistent wear helps improve masticatory muscle coordination over time [41]. Clinical management of discomfort includes recommending mild analgesics during the early phase, advising a soft diet for the first 24-48 hours, smoothing or coating hooks and providing wax where needed, selecting an appropriately sized elastic to reduce friction, and switching to non-latex alternatives if signs of hypersensitivity appear. Breakage is most often related to overstretching or extended wear beyond the recommended duration, and can be minimised by advising twice-daily replacement of elastics [42].

Integrating Compliance Into Clinical Practice

Compliance with IMEs can be improved through a combination of behavioural, educational, and comfort-focused strategies. Chairside demonstration followed by patient repetition helps ensure correct placement technique, while establishing clear expectations regarding daily wear and treatment objectives enhances accountability. Motivational reminders, whether digital or verbal, have been shown to reinforce routine use [36,37], and empathetic communication between clinician and patient strengthens trust and promotes cooperation [39]. Parental supervision remains particularly valuable when treating younger patients, and effective management of discomfort during the initial week can reduce early dropout. Positive reinforcement further supports the development of consistent habits. When these strategies are communicated effectively and reinforced consistently, elastics shift from a compliance-sensitive auxiliary to a reliable, well-integrated component of orthodontic treatment.

Comparison between IMEs and fixed functional appliances (FFAs)

IMEs and FFAs are widely used to correct Class II malocclusions. Although both approaches aim to improve sagittal relationships, they differ significantly in biomechanics, dependence on patient cooperation, skeletal and dentoalveolar effects, and overall treatment efficiency.

Treatment Efficiency and Duration

FFAs apply a continuous, non-removable force and therefore do not rely on patient compliance for effectiveness. Forsus appliances typically achieve Class II correction in approximately 4.5 months, whereas IMEs often require closer to seven months even when worn consistently [43]. This difference in treatment duration is driven primarily by variability in patient compliance rather than by any mechanical limitation of the elastic systems themselves.

Dependence on Patient Cooperation

IMEs are removable appliances and, therefore, highly dependent on patient adherence, whereas FFAs exert force continuously and effectively eliminate compliance as a variable [1]. Consequently, IMEs are most appropriate for demonstrably cooperative patients, while FFAs are generally preferred in situations where patient compliance is uncertain or predictably poor.

Skeletal and Dentoalveolar Effects

The literature indicates that both IME and FFAs correct Class II malocclusions predominantly through dentoalveolar changes rather than skeletal modification. Janson et al. (2013) found similar long-term outcomes between IME and FFAs, with both modalities producing mainly dentoalveolar changes [44]. Rigid FFAs may produce slightly greater skeletal effects than elastics, while flexible or hybrid FFAs mimic the dentoalveolar pattern of IME [45]. Thus, in most clinical scenarios, FFAs and IME offer functionally comparable biomechanical outcomes, differing mainly in their mode of delivery and reliance on cooperation.

Facial Profile and Aesthetic Outcomes

Soft-tissue enhancement is an important objective in the correction of Class II malocclusion. Current evidence indicates that both IME and the Twin Force Bite Corrector Device made by Ortho Organizers, Inc. (Henry Schein, Inc., Melville, New York, United States) contribute to improvements in facial profile attractiveness. However, treatment with the Twin Force Bite Corrector appears to produce a slightly greater reduction in facial convexity, suggesting a modest advantage in skeletal balance [46]. Janson et al. (2013) further noted that the soft-tissue effects of IME require additional investigation, as most existing studies have focused primarily on dentoalveolar changes rather than detailed facial aesthetic outcomes [44].

Vertical and Dental Side Effects

Differences in vertical control between IMEs and FFAs are clinically important. IMEs are associated with a tendency toward maxillary incisor extrusion and clockwise rotation of the occlusal plane, effects that can be undesirable in patients who are already vertically sensitive. In contrast, FFAs generally provide superior vertical control, frequently producing mandibular incisor intrusion and demonstrating less alteration of the occlusal plane [43]. These characteristics make FFAs particularly advantageous in high-angle cases where careful management of vertical dimension is essential.

Indications for Each Modality

IMEs are most appropriate for patients who demonstrate reliable cooperation, particularly in cases of mild to moderate Class II discrepancies where fine-tuned biomechanical control is desirable. They also offer a cost-effective option for individuals who can maintain consistent wear. In contrast, FFAs are better suited for patients with poor or unpredictable compliance, for moderate to severe skeletal Class II malocclusions, and for situations requiring faster sagittal correction. FFAs are also advantageous in high-angle patients, where superior vertical control is essential.

Conceptual Comparison

**Figure 5:**
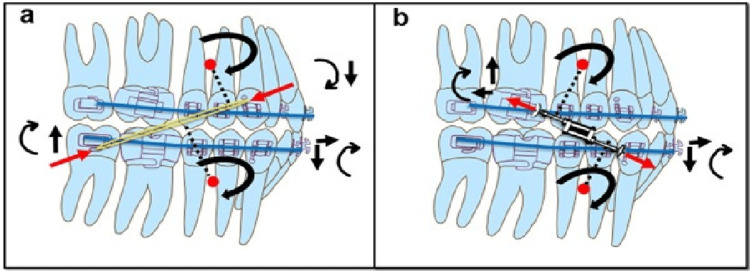
Comparative biomechanical effects of Class II elastics and the Twin Force® Bite Corrector (A) Class II elastics, in yellow, exert an oblique, pull mechanics, force vector extending from the maxillary canine to the mandibular molar, passing above the mandibular and below the maxillary centers of resistance (red dots). This produces clockwise rotation of both arches (black curved arrows), and proclination of mandibular incisors. (B) The Twin Force® Bite Corrector generates a similar oblique vector but with push mechanics and with greater and continuous force magnitude. The resulting pattern of movement mirrors that of Class II elastics, though with increased lower incisor proclination. Red arrows indicate the direction of force; black arrows indicate the direction of tooth movement; curved arrows denote rotational moments; red dots mark the centers of resistance; and dotted lines mark the distance from CR to line of force. CR: centre of resistance image Credit: Authors

Class II elastics and the Twin Force Bite Corrector generate broadly similar intermaxillary oblique vectors, but the resulting force systems differ in magnitude and continuity. Fixed functional appliances deliver greater and uninterrupted force, producing movement patterns comparable to those generated by elastics yet often accompanied by increased proclination of the lower incisors and more predictable sagittal correction. These contrasting biomechanical effects are depicted in Figure 5.

Summary of Key Considerations

IME and FFAs tend to produce broadly comparable dentoalveolar changes over the long term; however, FFAs generally achieve these effects more rapidly, do not rely on patient compliance, and offer superior vertical control. IMEs, by contrast, remain a highly versatile, cost-effective option when patient cooperation is dependable. Ultimately, the choice between these modalities should be guided by the patient’s skeletal pattern, expected level of compliance, and the degree of vertical control required.

Complications related to intraoral orthodontic elastics (IOEs)

Complications associated with IOEs arise from their inherent mechanical behaviour as well as errors in patient use or clinical supervision. These complications range from benign inefficiencies to significant biological damage. Understanding the mechanisms behind these adverse events is essential for risk minimisation and safe clinical practice.

Mechanical Instability and Force Degradation

All types of IOEs, whether latex or non-latex, undergo rapid force degradation, particularly within the first three hours of wear [47]. Latex elastics generally demonstrate greater force stability and retain a higher proportion of their initial force compared with non-latex alternatives [48,49], whereas non-latex elastics tend to exhibit more pronounced and continuous degradation, necessitating more frequent replacement to maintain effective loading. Several external factors further accelerate this decline: exposure to artificial saliva increases water absorption and polymer relaxation; cigarette smoke alters surface chemistry and mechanical properties [23]; and electromagnetic radiation has been shown in vitro to adversely affect elastic strength [24]. Such mechanical instability compromises the predictability of tooth movement and may result in delayed or incomplete correction if elastics are not replaced regularly.

Clinical Misuse and Poor Supervision

Several significant complications can arise when elastics are placed incorrectly, used without supervision, or retained intraorally for extended periods. Documented adverse events include tooth loss caused by a displaced elastic acting as a ligature around the gingiva or root [50], the development of intrabony defects when elastics become embedded within the periodontal apparatus [51], and long-term gingival embedding of retained elastics, in some cases persisting unnoticed for years [52]. Such complications typically occur when elastics migrate subgingivally and remain undetected, underscoring the importance of providing clear patient instructions, ensuring regular follow-up monitoring, and strictly avoiding any unsupervised or non-orthodontic use of elastics. Ultimately, misuse of elastics represents the most preventable source of serious adverse outcomes.

Clinical Implications and Risk Minimisation

Given the mechanical degradation of IOEs and the potential for iatrogenic injury, several measures are essential to ensure their safe use. Elastics should be replaced twice daily to counteract early force decay and maintain biologically effective tension, and their use should be closely supervised, particularly in younger patients or those with inconsistent compliance. Patients must be educated on correct placement and made aware of the risks associated with off-label or unsupervised use, while prolonged wear of fatigued elastics should be avoided to reduce the likelihood of irritation or subgingival migration. Routine monitoring of soft tissue health during orthodontic appointments is also important for detecting early signs of irritation or embedded material. When appropriately prescribed, correctly used, and carefully monitored, elastics remain a safe and effective adjunct in orthodontic treatment; most complications arise from misuse or inadequate follow-up rather than from the properties of the elastic material itself.

The triad of success in orthodontic elastic therapy

**Figure 6:**
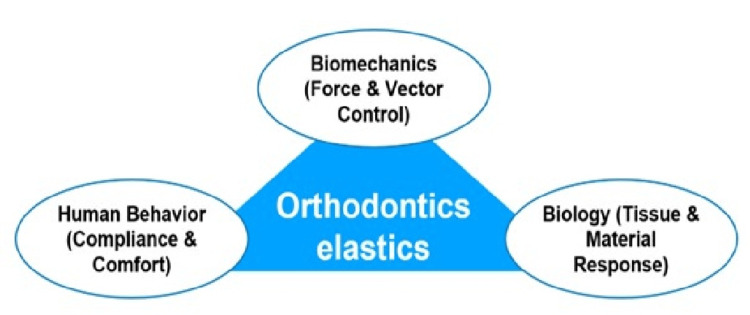
The triad of success in orthodontic elastic therapy A conceptual model illustrating the three interconnected domains required for predictable elastic therapy: biomechanics (force direction and vector planning), biology (material properties and force decay), and patient behaviour (compliance and correct use). All three elements must function together for optimal clinical outcomes. Image Credit: Authors

The predictability of orthodontic elastic therapy relies on the coordinated interaction of biomechanics, biology, and patient behaviour, which together form an integrated therapeutic framework. From a biomechanical perspective, the magnitude, direction, and line of action of the applied force must be carefully planned, with appropriate configuration and anchorage selection to achieve controlled tooth movement while limiting undesirable vertical or transverse effects. The biological domain influences how these forces are expressed over time, as material properties such as elastic memory and force-decay behaviour determine whether physiologically effective forces can be maintained throughout wear. Ultimately, patient behaviour becomes the decisive element, as consistent full-time wear, correct placement, and adherence to replacement protocols are necessary for converting biomechanical design into actual clinical movement. The success of elastic therapy, therefore, depends not on mechanics alone, but on the balanced alignment of force planning, material performance, and patient cooperation, as illustrated in Figure 6.

## Conclusions

Orthodontic elastics remain a foundational element of contemporary orthodontic biomechanics, offering a versatile and effective means of generating controlled tooth movement in both fixed appliance and aligner-based therapies. Their success depends on the coordinated interaction of biomechanical design, biological force expression, and patient behaviour. Latex elastics continue to demonstrate superior mechanical characteristics, particularly in terms of force stability and elastic memory. In contrast, non-latex alternatives provide essential options for patients with hypersensitivity or for clinical situations requiring prolonged wear. Optimal outcomes rely on precise control of force vectors, careful management of vertical and transverse side effects, appropriate anchorage planning, and frequent elastic replacement to counteract rapid intraoral force decay. Integration with skeletal anchorage and digital orthodontic systems further enhances vector precision and expands the scope of elastic-based treatment applications.

Equally central to clinical success is the patient-centred dimension of elastic therapy. Consistent full-time wear, correct placement, and adherence to replacement protocols determine whether intended biomechanical effects are translated into predictable clinical change. Effective communication, behavioural reinforcement, and routine monitoring can transform elastics from a compliance-sensitive auxiliary into a reliable, high-value component of treatment. Future developments should include improved non-latex formulations with enhanced mechanical stability, digital tools for monitoring and supporting compliance, and further refinement of elastic biomechanics within aligner systems. As orthodontics moves toward increasingly individualised and precision-focused care, a deep understanding of elastic biomechanics, biological response, and patient engagement will remain integral to achieving safe, efficient, and evidence-based outcomes.
